# Maximising the health impacts of free advice services in the UK: A mixed methods systematic review

**DOI:** 10.1111/hsc.13777

**Published:** 2022-03-21

**Authors:** David Young, Geoff Bates

**Affiliations:** ^1^ 1555 Sustainable Housing and Urban Studies Unit (SHUSU) University of Salford Salford UK; ^2^ 1555 Institute for Policy Research University of Bath Bath UK

**Keywords:** advice services, health, wellbeing

## Abstract

After a decade of austerity spending cuts and welfare reform, the COVID‐19 pandemic has posed further challenges to the finances, health and wellbeing of working‐age, low‐income people. While advice services have been widely seen (and funded) as an income maximisation intervention, their health and well‐being impact is less clear. Previous systematic reviews investigating the link between advice services and health outcomes have found a weak evidence base and cover the period up until 2010. This mixed methods review examined up to date evidence to help understand the health impacts of free and independent welfare rights advice services. We included evaluations of free to access advice services on social welfare issues for members of the public that included health outcomes. Through comprehensive searches of two bibliographic databases and websites of relevant organisations we identified 15 articles based on a mixture of study designs. The advice interventions evaluated were based in a range of settings and only limited information was available on the delivery and nature of advice offered. We undertook a convergent synthesis to analyse data on the effectiveness of advice services on health outcomes and to explain variation in these outcomes. Our synthesis suggested that improvements in mental health and well‐being measures are commonly attributed to advice service interventions. However, there is little insight to explain these impacts or to inform the delivery of services that maximise health benefits. Co‐locating services in health settings appears promising and embracing models of delivery that promote collaboration between organisations tackling the social determinants of health may help to address the inherent complexities in the delivery of advice services and client needs. We make recommendations to improve routine monitoring and reporting by advice services, and methods of evaluation that will better account for complexity and context.

**What is known about the topic**

- Advice services have been shown to address poverty through income maximisation.
- There is evidence of improvements in mental health and wellbeing linked to advice services.
- The association between advice services and health outcomes is complex and not well evidenced.

**What the paper adds**

- This systematic review synthesises limited evidence from 2010 to 2020 that suggests a positive association between advice services and health and wellbeing.
- Our mixed methods review approach highlights how our understanding of this association is limited by an inconsistent evidence base that lacks outcomes focusing on service implementation and delivery, or the experiences of clients or staff.
- We make recommendations for routine monitoring and comprehensive evaluative studies to better account for complexity and context.

## 1 INTRODUCTION

The COVID‐19 pandemic has drawn attention to existing and widening health inequalities and how they can be addressed. It has also led to an unprecedented demand for social security benefits and welfare rights advice services. At the start of the first lockdown period in March 2020, there was a significant increase in the number of people claiming Universal Credit (HOC Library, 2020) as well as various government support packages (Hick & Murphy, 2020). There was also a spike in the need for information and advice on social welfare issues (Citizens Advice, 2020, p. 14). Advice services within this review are defined as services that are providing free and independent advice on social welfare issues, available to the general population in the UK such as Citizens Advice services (Citizens Advice, 2021) and meeting official advice quality standards (Advice Services Alliance, 2021). Social Welfare issues are defined by Advice UK as: ‘Areas of civil law relevant to difficulties most frequently experienced by people who are on low incomes or who are otherwise disadvantaged. Areas of social welfare law and advice include (but are not restricted to): debt, welfare benefits, housing, employment, education, discrimination, immigration, community care and consumer rights’ (Advice UK, 2021, p. 1).

The impact of low income and poverty on health is well documented (Benzeval et al., 2014; Marmot, 2020) and within this context, advice services have been seen as interventions that can address low income and poverty. However, the link between advice service interventions and health and wellbeing is less clear within the literature. While there is a body of evidence that has identified the mental health and well‐being impacts of advice interventions (Citizens Advice, 2012), these links have proved hard to evaluate due to the complexity of interventions, advice populations and health and well‐being outcomes (Abbott, 2002; Adams et al., 2006; Allmark et al., 2013).

Previous systematic reviews on this topic have been carried out but only review evidence up to 2010. Adams et al. (2006) address the question ‘what are the health, social and financial impacts of welfare rights advice delivered in healthcare settings?’. They find that although there were good theoretical reasons why advice improved health and wellbeing, there was a lack of robust evidence that the impact of advice goes beyond income gains. A more recent review of the literature using systematic review principles was Allmark et al. (2013) who address the question ‘what are the elements in a causal pathway between advice intervention and health outcomes?’ by reviewing literature published in English up to February 2010. They use a logic model to set out the complex links between advice interventions and health and well‐being outcomes drawing on a wider range of evidence. Both studies identify a complex picture with well‐evidenced financial benefits of advice but a lack of good quality and consistent evidence of the impact of advice services on health and well‐being outcomes in the period to 2010. In addition to these systematic reviews, a recent scoping review of related evidence focused on the health–justice partnerships in healthcare settings. Beardon et al. (2021) found that Health Justice Partnerships around the world effectively address the social welfare issues that affect health. Our review complements this study by focusing on the wider range of settings and services outside of health justice partnerships in the UK context. In addition to focusing on the effectiveness of services, we are concerned with how these services are delivered and how they are evaluated.

The decade between 2010 and 2020 has been a period of significant change, particularly for those of working age who engage with the social security system. A period of austerity adopted as an economic response to the crash of 2008 meant unprecedented public sector cuts and widespread policy change in a process of welfare reform (Millar & Sainsbury, 2018). At the end of this decade, the COVID‐19 pandemic has drawn attention to vast inequalities within the UK population. The social, economic and health impacts of the pandemic have been experienced unequally, with deprived communities and people from Black, Asian and Minority Ethnic backgrounds more likely to have their health affected and to already be experiencing poor housing, poor employment conditions and poverty (Cheater, 2020). In addition, there has been a greater need for help accessing new forms of financial support such as the Coronavirus Job Retention Scheme (CJRS) and the Self Employment Income Support Scheme (SEISS), and to understand changes in benefit and housing regulations (Brewer & Gardiner, 2020). These complex and interacting inequalities show how interlinked health, housing, employment and financial security are in people’s lives and raises the question of how advice services can address them.

It has also drawn attention to the role advice services can play within a context of increasing statutory emphasis on holistic approaches to health and wellbeing, including the requirement of a local authority plan for advice provision under the Care Act (2014). Clinical Commissioning Groups (CCGs) also fund advice services in specific areas, but this approach is inconsistent across the country. In order for commissioners to assess the effectiveness of such interventions and to support the optimal delivery of services on health and well‐being outcomes, updated evidence is needed.

This mixed methods review updates this body of evidence to 2021 to cover a period of unprecedented economic, social and policy change. There is a pressing need to firstly understand the impact of advice services in a tough funding environment, and secondly to understand what we know about the relationship between advice interventions and health outcomes, and what more we need to know. Therefore, the review seeks to identify and understand the health impacts of advice services to make recommendations for advice providers, policy makers and funders. It synthesises evidence on health and well‐being impacts from quantitative and qualitative evaluations of advice services delivered by not for profit or public sector organisations in the UK to understand the impacts of services and factors that may explain these impacts. The development of the review was informed by a protocol (available at: www.bath.ac.uk/projects/advice‐services‐and‐health‐outcomes/).

## 2 METHODS

The review methods were adapted from the Joanna Briggs Institute’s process for undertaking mixed methods systematic reviews (JBI, 2014). The PRISMA statement (Page et al., 2021) informed the development of methods and reporting of review processes and the completed PRISMA checklist for this review is provided in section six of the supplementary materials.

### 2.1 Inclusion criteria

All free to access services giving advice on social welfare issues, delivered by public sector or not for profit organisations were eligible for inclusion. This has been defined as services that are providing free, independent advice on social welfare issues available to the general population in the UK and meeting official advice quality standards (Advice Services Alliance, 2021).

This included citizens advice and equivalent services offered by other independent organisations and through local authorities; services delivered by volunteers or professionals in face to face, telephone or online formats using a drop in or appointment‐based approach; based in any setting; and that were accessible to anyone. A range of interventions are delivered in advice services. This includes those such as: generalist and specialist advice on a range of social and welfare issues such as welfare benefits, housing, debt and employment; referral and signposting to other services. All interventions types were included (see Welsh Government, 2013 for a comprehensive list of advice service interventions). Health advice or services offering health interventions only were excluded, for example stop smoking services.

Studies that included health outcomes of any clients accessing services were eligible for inclusion. Identification of health outcomes was informed by a logic model created by Allmark et al. (2013) that projected factors leading to changes in health status following access to advice services and included any measures of: physical health status, mental health conditions, well‐being indicators, use of healthcare services and health behaviours (such as smoking, physical activity and diet).

Amongst studies that included health and well‐being outcomes, we additionally sought any measure of service implementation or delivery to help understand health impacts, including satisfaction, barriers and enablers, accessibility, or acceptability. Outcomes for both clients and service providers were included. Finally, we included client outcomes reporting the social determinants of health (as identified in the logic model developed by Allmark et al. (2013) and Dahlgren and Whitehead’s (1991) social determinants of health model. These included housing, employment, working conditions, access to healthcare, education, food, social relationships and home environment.

We included evaluations of advice services based on any study design. All studies that evaluated relevant advice services using any study design, including either or both quantitative or qualitative designs, were eligible for inclusion.

### 2.2 Search strategy

A comprehensive search was undertaken between December 2020 and January 2021 to identify studies in the academic and grey literatures that were published in English. The most recent previous review of the health impacts of advice services (Allmark et al., 2013) included studies published up to 2010 and therefore we included literature published from January 2010. Following a scoping search in Medline to identify key search terms we developed a search strategy to search within the Medline and Social Policy and Practice bibliographic databases. The search strategy used in Medline is reported in the online supplementary materials.

To identify grey literature, we compiled a comprehensive list of key organisations (included in the supplementary materials) and searched their online publication lists and libraries. The reference lists of all studies included in the review and related literature reviews identified in our search were scanned to identify any additional articles.

### 2.3 Study selection

Academic literature search results from the two databases were exported to Endnote, combined and duplicates deleted. One reviewer screened titles and abstracts of all studies identified through the database and grey literature searches against the review inclusion and exclusion criteria. A 10% sample of these studies was independently checked by a second reviewer, with any differences in interpretation resolved through discussion.

### 2.4 Data extraction and quality assessment

Data extraction and quality assessment were carried out by one reviewer with 10% independently carried out by a second reviewer. Differences in interpretation were resolved through discussion. Data were extracted into a pre‐designed form in Microsoft Access including study characteristics; the characteristics of participants, interventions, and settings; outcomes; and study limitations.

Assessment of methodological quality was challenging due to the range of study designs used and the inclusion of studies from the grey literature that did not necessarily provide the level of detail or methodological rigour associated with academic articles. The quality of studies where sufficient detail was provided for rigorous assessment was determined using the JBI tools for critically appraising quasi‐experimental and qualitative study designs (JBI, 2020a, 2020b). Based on the findings of quality assessment, the strength of evidence was rated ‘strong’ (>81% yes answers to JBI criteria), ‘moderate’ (>61% yes answers to JBI criteria) or ‘weak’ (60% and fewer yes answers to JBI criteria). For all studies where quality assessment was not feasible, strength of evidence was assessed as ‘un‐rated’.

### 2.5 Synthesis

We adopted a mixed methods approach to synthesise data from all study designs and by outcomes. The lack of high‐quality quantitative studies influenced this approach and limited the role of quantitative data. A convergent segregated design (Aromataris & Munn, 2020) allowed us to independently synthesise qualitative and quantitative data before integrating them in tabular and narrative summaries. Review findings are presented in a narrative synthesis and in structured tables. These tables present findings by outcome type and highlight the strength of evidence for each outcome (see Tables 1 and 2). Study findings were grouped by outcome type into three overarching outcome categories: health, determinants of health and service delivery and implementation. Within each overarching category, we grouped similar measures into subcategories and assessed the overall strength of evidence within each outcome subcategory according to the methodological strength of evidence, consistency in the direction of evidence, and amount of evidence available.

**TABLE 1:** Summary of included articles

Author (Methodological quality)	Intervention(s) provided	Summary of approach	Outcomes measured
Boston Citizens Advice (2012) (Un‐rated)	Advice on prescription service providing comprehensive benefits advice and help with applications and appeals	Evaluation of service health and financial impacts based upon analysis of case files and client survey	**Health**: wellbeing measures; **financial**: income gains
Burrows et al. (2011) (Moderate)	Welfare advice in primary care on any issue	Qualitative evaluation examining views and experiences of service staff (*n* = 22) and users (*n* = 12).	**Health**: mental health conditions; **financial**: income gains, debt managed; **service implementation and delivery**: accessibility
Cooper (2015) (Weak)	Information and advice on housing, care and finance for older people.	Evaluation of an advice service based on review of service data, case studies and interviews with clients (*n* = 44) and staff and stakeholders (*n* = 21)	**Health**: wellbeing measures; **financial**: income gains, debts managed; **service implementation and delivery**: satisfaction.
Dalkin et al. (2019) (Moderate)	Advice on many issues including benefits, housing, employment and debt	Realist evaluation based on survey data (*n* = 191) and interviews (*n* = 22) seeking to understand the role of an advice service on stress and wellbeing.	**Health**: well‐being measures (WEMWBS), stress (PSS)
East Staff CAB (2015) (Un‐rated)	Advice on a range of issues (including benefits, debt, housing and employment).	Service evaluated based on service data and client satisfaction survey (*n*= not reported).	**Health**: mental health conditions, well‐being measures, stress; **financial**: income gains, debts managed; **service implementation and delivery**: satisfaction.
Farr et al. (2014) (Moderate)	Advice on a wide range of issues including employment disputes, housing, debt and benefits.	Mixed methods evaluation of service impacts based on case files and interviews with clients (*n* = 80) who were followed up (*n* = 38) in periods of up to 2 years.	**Health**: well‐being measures (WEMWBS); **financial**: income gains.
Howel et al. (2019) (Strong)	Domiciliary welfare rights advice consultations and active assistance with benefit claims for older people.	Mixed methods evaluation (RCT, economic evaluation and process evaluation) exploring service impacts on clients (*n* = 562) quality of life.	**Health**: well‐being measures (CASP−19)
Jones (2011) (Moderate)	Advice on a wide range of issues including employment disputes, housing, debt, benefits and relationships.	Mixed methods evaluation of service health and financial impacts based on longitudinal survey and interviews (at baseline (*n* = 149), 6‐month follow up (*n* = 76) and post−12 month follow up (*n* = 42))	**Health**: mental health conditions (HADS), well‐being measures (SF−36); **financial**: income gains, debts managed
Kerr et al., (2019) (Weak)	Advice on prescription service in primary care settings, including income maximisation to ensure all eligible income is secured.	Service evaluation based on review of service data and client interviews (*n* = 13)	**Health**: well‐being measures; **financial**: income gains, debts managed
Krska et al. (2013) (Moderate)	Citizens Advice Bureau health outreach in primary care services	Mixed methods evaluation based on interviews with staff in advice and GP services and analysis of medical records, to understand staff experiences and use of healthcare services.	**Health**: use of healthcare
NHS Sefton (2010) (Moderate)			**Health**: use of healthcare; **service implementation and delivery**: satisfaction
Moffatt et al. (2010) (Moderate)	Full welfare benefits check followed by assistance to claim entitlements, follow‐up work and representation for people affected by cancer	Qualitative evaluation based on interviews with clients and their carers (*n* = 22) to understand health impacts of the service.	**Health**: wellbeing measures, stress
Moffatt et al. (2012) (Moderate)	Mixed methods evaluation that uses casefiles to assess the welfare outcomes among 533 male and 641 female cancer patients and carers; and qualitative interviews with patients (*n* = 35) and carers (*n* = 9).	**Health**: mental health conditions, wellbeing measures, stress; **financial**: income gain
Woodhead et al. (2017) (Strong)	Co‐located welfare rights advice in primary care	Prospective quasi‐experimental controlled study of the impact of advice services (*n* = 8 intervention & *n* = 9 comparator sites) on common mental health disorders and income.	**Health**: mental health conditions (GMQ−12), well‐being measures (SWEMWBS), stress; **financial**: income gains; **service implementation and delivery**: accessibility
Woodhead et al. (2017) (Strong)		Mixed methods evaluation of service impacts including interviews with GPs and advisers (*n* = 24) and surveys (*n* = 278 advice clients; 633 controls)	

Note

Quality ratings: Strong (>81% Y’s on JBI checklist), Moderate (>61% Y’s on JBI checklist), Weak (60% and below Y’s on JBI checklist), Un‐rated (not enough methodological detail to assess)

**TABLE 2:** Summary of health outcomes

Citation	Methodological quality	Health and wellbeing outcomes			
		Mental health	Wellbeing	Stress	Use of healthcare
Boston Citizens Advice (2012)	Un‐rated	N/A	⇧	N/A	N/A
Burrows et al. (2011)	Moderate	⇧	N/A	N/A	N/A
Cooper (2015)	Weak	N/A	⇧	N/A	N/A
Dalkin et al. (2019)	Moderate	N/A	⇧	⇧	N/A
East Staff CAB (2015)	Un‐rated	⇧	⇧	⇧	N/A
Farr et al. (2014)	Moderate	N/A	⇧	N/A	N/A
Jones (2011)	Moderate	⇧	⇧	⇧	N/A
Howel et al. (2019)	Strong	N/A	⇔	N/A	N/A
Kerr et al., (2019)	Weak	N/A	⇧	N/A	N/A
Krska et al. (2013)	Moderate	N/A	N/A	N/A	⇕
NHS Sefton (2010)	Moderate	N/A	N/A	N/A	⇕
Moffatt et al. (2010)	Moderate	N/A	⇧	⇧	N/A
Moffatt et al. (2012)	Moderate	⇧	⇧	⇧	N/A
Woodhead, Djuretic et al. (2017) and Woodhead, Khondoker et al. (2017)	Strong	⇧	⇕	⇧	⇕

Note

⇧positive impact; ⇕mixed impacts across measures (combination of positive, negative or no impact); ⇔no impact; N/A not applicable

## 3 RESULTS

**FIGURE 1:**
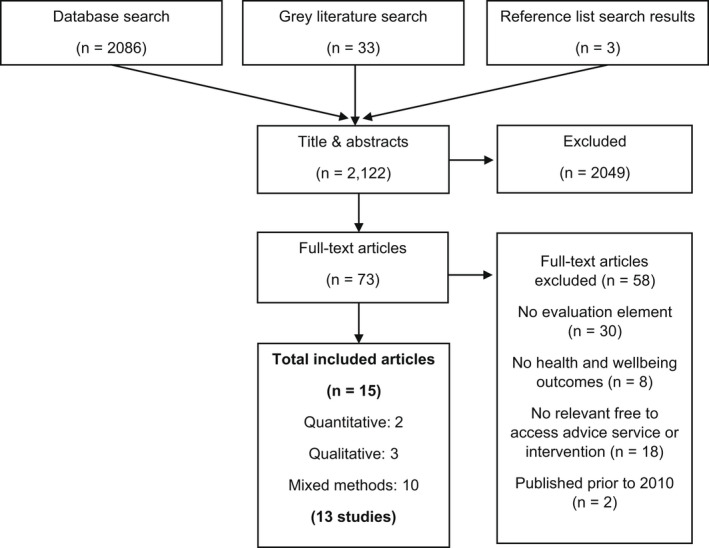
Flow chart of study selection

Following the study selection process, we included 13 studies evaluating the impacts of advice services on health outcomes, reported in 15 articles. The flow of articles through the review is presented in Figure 1. Reasons for study exclusion at full text assessment stage are reported in full in the online supplementary materials.

### 3.1 Summary of identified articles

A summary of the included studies is provided in Table 1. Service setting varied with six articles (four studies) focusing on advice provided in healthcare settings (Burrows et al., 2011; Kerr et al., 2019; Krska et al., 2013; NHS Sefton, 2010; Woodhead, Djuretic et al., 2017; Woodhead, Khondoker et al., 2017), three articles on advice office settings (Boston Citizens Advice, 2012; Dalkin et al., 2019; East Staffordshire CAB, 2015), one article on domiciliary advice provision (Howel et al., 2019) and five articles (four studies) on advice provided in various settings (Cooper, 2015; Farr et al., 2014; Jones, 2011; Moffatt et al., 2010, 2012). In addition to services provided to the general population, studies evaluated the impact of advice provided to cancer patients (Moffatt et al., 2010, 2012) and older adults aged over 60 (Howel et al., 2019). Advice services typically offer advice and information covering a wide range of social welfare issues, such as finances, employment, housing and benefits. Typically, the services evaluated here provided advice covering some or all of these topics, but there was very limited detail reported on the nature of the interventions. Additionally, there was little detail on the content or delivery of advice. Studies used a range of outcome measures to evaluate the impact of services on predominantly mental health and well‐being outcomes, while outcomes relating to determinants of health were predominantly financial outcomes.

### 3.2 Study quality

The inclusion of all study types in order to gather a wide body of evidence meant a diverse range of studies. In order to understand the evidence better and to consider what confidence we had in each piece of evidence, we critically appraised it using JBI appraisal criteria (JBI, 2021). Eleven studies provided enough information for rigorous appraisal and two studies did not (Boston Citizens Advice, 2012; East Staffordshire CAB). The studies that provided enough information for rigorous appraisal generally met the criteria set out in the JBI quasi‐experimental or qualitative tools and studies scored highly. The overall assessments of study quality are included in Table 1, and detail on the application of the JBI tools to each study is provided in sections three and four of the supplementary materials. While the majority of studies included were rated moderate or strong for methodological quality, overall the strength of the evidence base was limited by diversity in outcome measures and reporting of intervention content, context and delivery. This restricted the potential to compare outcomes across studies and to understand what types of services and context are associated with different health impacts.

Quasi‐experimental studies clearly set out cause and effect, used multiple outcome measures pre and post intervention, documented details of follow ups, measured outcomes in a reliable way and used appropriate statistical analysis. A control group was used in two of three studies, but it was unclear whether comparison groups were receiving similar treatment/care. In the qualitative studies, we identified consistency within each study between the stated philosophical and methodological perspectives, and the research design and methods applied. While detail on ethical approval was included in all but two studies (Cooper, 2015; Kerr et al., 2019), there was a lack of detail of the influence of the researcher on the research, including a lack of statements locating the researcher culturally or theoretically. Study limitations were generally well set out and explained but revealed the complexity of evaluations and the difficulty of applying scientific methods to complex social environments. Attributing health and well‐being outcomes to advice interventions was reported as challenging by study authors (For example: Farr et al., 2014, p. 64). Two service evaluations based on case files and satisfaction carried out by advice services (Boston Citizens Advice, 2012; East Staffordshire CAB, 2015) could not be assessed using the JBI tools because of a lack of information or an apparent lack of methodological consideration.

### 3.3 The impact of advice services on health

All included studies evaluated the impact of advice services on health including mental health conditions, wellbeing, stress and use of healthcare. Findings indicate that advice services have typically been associated with positive impacts on health outcomes (Table 2), but the overall strength of the evidence limits our confidence in these results. The direction of evidence must therefore be considered in the context of the limitations of the evidence base with substantial variation in outcome measures and evaluation methods. Additional information on the specific outcome measures used and detail on the findings from individual studies are included in the supplementary materials.

Overall, our greatest confidence is in the evidence of mental health and stress outcomes, where studies of predominantly strong or moderate methodological quality reported consistently positive outcomes. Five studies (six articles) of mixed quality reported positive mental health condition impacts (Burrows et al., 2011; East Staffordshire CAB, 2015; Jones, 2011; Moffatt et al., 2012; Woodhead, Djuretic et al., 2017; Woodhead, Khondoker et al., 2017). One study found that advice addressed the symptoms of mental illness (Burrows et al., 2011), one study (two articles) found a reduction in the proportion of individuals meeting the criteria for common mental disorders (CMD) in those receiving advice compared with a control (Woodhead, Djuretic et al., 2017; Woodhead, Khondoker et al., 2017), and three studies found reductions in anxiety and depression that they attributed to advice (East Staffordshire CAB, 2015; Jones, 2011; Moffatt et al., 2012).

Similarly, six studies (seven articles) of mixed quality reported positive impacts on client stress, including reduced stress and better coping skills (Dalkin et al., 2019; East Staffordshire CAB, 2015; Jones, 2011; Moffatt et al., 2010, 2012; Woodhead, Djuretic et al., 2017; Woodhead, Khondoker et al., 2017). However, the evidence‐base remains small for these outcomes (reduced stress and better coping skills) and while understanding of well‐being impacts is based upon a larger number of studies (*N* = 10), this included studies of weaker methodological quality. Additionally, while the majority indicated positive well‐being impacts, it is noted that stronger quality studies identified some inconclusive effects (Howel et al., 2019; Woodhead, Djuretic et al., 2017; Woodhead, Khondoker et al., 2017). For example, in one study it was reported that there was no difference in change of wellbeing between participants who received advice and controls, but subgroup analysis indicated that a positive impact on other outcomes with receiving advice was associated with improved wellbeing (Woodhead, Djuretic et al., 2017). This highlights an area of complexity in analysing the impact of these services. Impact of receiving advice on use of healthcare was inconclusive with evaluation of this outcome limited to two studies (four articles) that indicated a mixture of positive, negative and no impact (Krska et al., 2013; NHS Sefton, 2010; Woodhead, Djuretic et al., 2017; Woodhead, Khondoker et al., 2017).

### 3.4 Determinants of health

Studies predominantly included financial outcomes as the only measures of the determinants of health. Three broad measures were used to determine financial outcomes: income gain, debts managed and written off, and other financial outcomes. The outcomes were suggestive of a positive impact of advice services. Detail on financial outcomes is provided in section five of the supplementary materials, and figures demonstrate the varying and inconsistent nature of financial outcome calculations within the included studies that makes comparison difficult. The range of figures used to show financial outcomes (From £92,000 to £6.7 million) and the varying contexts (local and national projects and differing time periods) again make it difficult to draw conclusions from these figures.

Three studies reported other outcomes, these were all housing related. East Staffordshire CAB (2015) identified 200 cases of homelessness being averted by their advice service in a financial year (in a caseload of 1067). Improved housing circumstances after advice were also reported by two other studies (Cooper, 2015; Woodhead, Khondoker et al., 2017).

### 3.5 Implementation and delivery

The studies included scant evidence on implementation outcomes or perceptions and experiences of using services. Positive outcomes were identified in six studies (seven articles) which related to service accessibility (*n* = 2) and satisfaction (*n* = 4).

In the studies reporting accessibility outcomes (Burrows et al., 2011; Woodhead, Djuretic et al., 2017), the location of advice services in GP surgeries was seen as increasing their accessibility. Woodhead, Khondoker et al. (2017) found that:

*“Nearly half of advice service users reported that had the service not been at the GP practice they would have gone to their GP for advice or would not have sought advice at all.” (p. 7)*

This was attributed to the familiarity and confidentiality of GP surgeries and the continuity of services. Burrows et al. (2011) also identified the importance of doctor referrals in increasing accessibility, as one advisor in their qualitative study explained:

A lot of people see the posters at GP surgeries but often it’s the GP who says specifically you need to go and get help with this. (p. 706)

Accessibility was also linked to the co‐location of advice services in GP surgeries making them easier to access, something reflected on by patients:

If you go to the doctors and you know there’s a Citizens Advice Bureau worker there, then you can just ask to make an appointment. (p. 707)

While only reported by two studies, the location of advice services appears to make a difference to accessing a service that has the potential to address low income and related health and well‐being outcomes. However, more focused research is needed.

In the studies reporting satisfaction outcomes (Cooper, 2015; East Staffordshire CAB, 2015; Kerr et al., 2019; NHS Sefton, 2010), these were all positive but were limited greatly by the limitations in study quality and reporting. It was not clear why clients were satisfied with services, or whether variation was explained by the context, content or delivery of interventions.

## 4 DISCUSSION

The period since 2010 in the UK has been one of unprecedented welfare reform alongside deep ‘austerity’ cuts that have had a disproportionate impact on low‐income working age people, including minority groups (Portes & Reed, 2018). Previously identified issues that advice services can help to address such as overstretched GP surgeries (Watt, 2011) and unequal access to support, information and resources are likely to have increased dramatically during the COVID‐19 pandemic. There has certainly been a dramatic increase in the number of people claiming means‐tested benefits (HOC Library, 2020) as well as an increase in the need for legal advice on social welfare issues (Citizens Advice, 2020; Newman et al., 2021). Additionally, the pandemic has disproportionately impacted those who are already at greater risk of suffering poor health outcomes and for whom advice services have a vital role. This includes evidence of increased risk of COVID‐19 infection and adverse impacts for ethnic minority groups (Kirby, 2020); those from lower socio‐economic backgrounds (Wright et al., 2020); those with long‐term health conditions (Huang et al., 2020; McQueenie et al., 2020; Sattar et al., 2020); and those living with disabilities (Bailey et al., 2021; Courtenay & Perera, 2020).

COVID‐19 reminds us of the complex relationship between welfare rights advice and health and highlights the need for accessible services that can have health and well‐being benefits for populations who suffer disproportionately poor health outcomes.

### 4.1 The health impacts of advice services

Despite the limitations in the evidence, there is a clear indication that advice services may contribute to positive wellbeing and mental health outcomes. This adds to evidence from previous reviews examining the association between advice and health (Adams et al., 2006; Allmark et al., 2013; Beardon et al., 2021) that supports the commissioning and delivery of advice services to improve public health, but highlights the weaknesses in the evidence base and our lack of understanding on mechanisms that drive this impact.

Our review suggests that there has been only limited progression of the evidence base in the past decade. Previous studies similarly have found a lack of good quality evidence linking advice interventions and health outcomes. This lack of evidence has meant that ‘…the rationale for implementing welfare advice as a health intervention is often left implicit’ (Dalkin et al., 2019, p. 768) and based on the assumption that tackling determinants of health will result in health and well‐being outcomes.

Indeed there are well‐established links between poverty, inequality and ill‐health in health research (Impact on Urban Health, 2021; Marmot, 2020). For example, there is evidence that money influences health (Benzeval et al., 2014; Leeds City Council, 2011), that debt problems are linked to poor health outcomes (Chew‐Graham, 2009; Fitch et al., 2011) and that financial capability can lead to improved longer term psychological wellbeing (Taylor, 2011). Income gains from welfare rights advice in healthcare settings have long been recorded and recognised. For example in their 2006 systematic review of the health, social and financial impacts of welfare rights advice in healthcare settings, Adams et al. (2006) identified a mean financial gain of £1,026 per client in the year following advice. It is likely therefore that the suggested health benefits can be largely explained through services addressing financial problems and other health determinants such as employment, housing and education; as well as through helping to reduce stress by providing an opportunity for clients to talk and be listened to. In their review of evidence up to 2010, Allmark et al. (2013) describe this as a counselling effect and the findings in our review support this idea with consistent reductions in stress reported following service attendance.

### 4.2 Implications for monitoring and evaluation

However, a substantial gap remains in our understanding of how such services can be optimally delivered so as to maximise these benefits. For example, we are unable to draw conclusions about specific interventions, methods of delivery, settings and target populations; all of which would support the commissioning and provision of cost‐effective services. Advice interventions are complex in nature and can involve substantial variation in settings, providers, content and delivery methods, and currently the evidence falls short of helping us to identify what types of interventions and models of service delivery are most effective, accessible and acceptable. The studies included in this review were of varying methodological quality and design; focused on a range of populations, settings and types of advice offered; and used different approaches to measure impact. To an extent this may reflect the variation in advice delivery and supports the need for evaluations to pay greater attention to context and variation in their design and reporting. Rigorous studies are needed that consider implementation and delivery of services as well as measuring health outcomes, and more consistent and robust monitoring activities that go beyond poorly explained income gains and satisfaction surveys. Improving the quality and consistency of routine monitoring and data collection activities across advice services will help to better demonstrate their health impacts and build convincing cases as they compete for scarce resources.

### 4.3 Locating advice services in healthcare settings

Due to the limitations and inconsistencies of the evidence base, it is challenging to draw firm conclusions about the most effective types of advice interventions and services. However, there is promising evidence suggesting that advice services can have a complimentary role when delivered alongside primary healthcare and that this improves service accessibility, limits the strain on primary healthcare resources and improves the take up and successful claiming of health related benefits (Woodhead, Djuretic et al., 2017, p. 6). This evidence builds on literature outside of the reviews scope, for example that which was published before 2010, that advice services in healthcare settings complement healthcare in a number of ways (Galvin et al., 2000; Harding et al., 2002; Moffatt et al., 2004; Paris & Player, 1993).

Co‐locating services in health settings specifically could have numerous advantages. In 2018, local Citizens Advice services were being delivered in 500 health locations such as GP surgeries (Budd, 2018, p. 2) with different funding arrangements. However, national coverage remains patchy (Low Commission, 2015, p. 74) and increasing the co‐delivery in primary healthcare of advice services is a possible avenue to improve the accessibility and cost‐effectiveness of services. It is estimated that around a fifth of problems dealt with by GPs in the UK are social rather than medical (Citizens Advice, 2015; Torjesen, 2016) and co‐location is likely to improve signposting and referral processes to support for these social problems. Citizens Advice also estimate that saving GP time, such as time spent on social problems that welfare rights advice services are well equipped to respond to, could lead to financial savings of up to £400 million a year for the NHS which they argue goes hand‐in‐hand with improving the overall health and wellbeing of patients (Budd, 2018; Caper & Plunkett, 2015). Including outcomes related to accessibility and client and provider satisfaction with different types of services and settings, and the experiences of healthcare professionals and impacts on healthcare in co‐delivered services more frequently within evaluations will provide important evidence to support our understanding of optimal co‐delivery of services.

Rather than framing advice services as standalone services, this review suggests that they should be seen as part of a number of complex interventions to address health and wellbeing. This has practical implications for how services are provided. While our review provides some specific evidence of the importance of co‐located advice services in primary care, more broadly it sets out a picture of complexity that could be addressed by a physical or virtual ‘hub’ approach to service delivery that includes an advice element. There are many, diverse examples of organisational collaborations of this nature within healthcare and community settings that have been referred to as ‘hub’ approaches, some of which involve the physical grouping of services to better address complex medical and support needs. This shared space and collaboration has been central to the COVID‐19 pandemic response of third sector organisations in specific areas of the country (Larkin et al., 2021). It can also be seen as sharing some of the key principles of social prescribing initiatives that bring together diverse practitioners to address wider social determinates of health (Social Prescribing Network, 2016).

### 4.4 Accounting for complexity and the wider system

Approaches to evaluate advice services need to better reflect the complex real‐world contexts that they exist in if we are to understand their impacts and to support the delivery of (cost)‐effective services to maximise health and well‐being benefits. Drawing further on mixed methods and qualitative approaches that seek to understand how services are experienced by those attending and delivering them, and to explore variation in outcomes will help to build a more complete picture of how advice services can improve health and wellbeing. Two studies included in this review were based on realist methods of evaluation.

Dalkin et al. (2019) test theories that explain the improved wellbeing of clients following advice and find increased client capabilities, the fostering of trusting relationships between client and adviser, and advice services creating a ‘third space’ between client and state (p. 773). Woodhead, Khondoker et al. (2017) focused on the importance of the healthcare setting of advice services and in particular how advice services can contribute to primary healthcare services such as GP surgeries. By seeking to understand how variation in context and mechanisms to bring about change are associated with variation in outcomes, future studies can start to better account for complexity in services and the context they are delivered in. Including measures of the implementation and delivery of services within evaluations is an important step in developing this evidence base.

Beyond the variation in advice services themselves, we must also consider the wider context that these services exist in. Support from advice services is likely to be just one factor that influences clients’ wellbeing at any one time within a complex system. Systems represent a group of interrelating and interacting components that directly or indirectly influence each other and systems‐based approaches to understand and change complex problems and behaviours reinforce that no part of the system is completely independent or exists in isolation (Lee et al., 2017; Michie et al., 2014). Therefore a change in one component will influence others and the combined influence of multiple components will be different from that of any individual component in isolation (Luke & Stamatakis, 2012). When tackling complex problems such as wellbeing and inequalities, public health research has increasingly embraced the idea of interventions such as advice services as ‘events’ within complex systems (Hawe et al., 2009; Moore et al., 2019; Peters, 2014).

Taking a systems perspective helps to understand complex problems and the impacts of interventions (Carey et al., 2015), and to account for this complexity in evaluations. For example provision of advice may have some short‐term health and well‐being benefits, but the extent of this impact may be mediated by other events in the system such as the accessibility of welfare support or the delivery of healthcare. While earlier studies have started to account for complexity in providing advice (Allmark et al., 2013; Dalkin et al., 2019) this evidence is still limited and adopting systems approaches to explore the impact of advice is an interesting avenue to take forward research in this area. Allmark et al. (2013) useful logic model to understand the health impacts of advice services could be extended to incorporate these systemic factors and influences, supported by activities such as actor mapping or systems mapping to gain a better understanding of the system that advice services sit within. These process can help to provide a wider perspective on a problem and to inform decision‐making amongst a range of stakeholders about the optimal ways to tackle complex problems on a local or wider stage (Egan et al., 2019).

### 4.5 Limitations

Our analysis found substantial inconsistency in the use of outcome measures in both advice service monitoring exercises and evaluative studies, which made comparison across studies challenging and prevents the development of a coherent evidence base on which to base our conclusions and recommendations. For example, wellbeing was measured using recognised scales such as the Warwick and Edinburgh Mental Wellbeing Scale (WEMWBS) (Farr et al., 2014; Forster et al., 2016) and general subjective measures derived from client survey questions (Boston Citizens Advice, 2012; East Staffordshire CAB, 2015). An important improvement would be an agreed set of measures that were implemented in different advice organisations across the country. Similarly, a limitation of the review is that we were unable to differentiate between the different types of advice and services represented in the studies that we included. Organisations and services also need to be clearer about what they are trying to achieve and what is being delivered. Interventions were described as advice services with little detail of their specific nature, while some went into detail about the services, they were evaluating such as an income maximisation service, others assumed an understanding of the diversity of advice on social welfare issues.

This review also excluded evaluations of wider interventions that included an advice element that could not be distinguished, such as social prescribing services. It would be beneficial to better understand the role of these wider interventions but clearly attributing health or well‐being improvements to advice alone is challenging. The review also excludes an assessment of Return on Investment (ROI) studies, although several of the included studies had an element of ROI. These were of varying form and quality and primarily show the need for services to evidence their ‘value’ in monetary terms. However, they perhaps also provide an example of how organisations explain complex connections between advice interventions and health outcomes.

Finally, a challenge for identifying this evidence was the varied terminology used by authors to describe the types of services and interventions we sought to identify. Additionally, we anticipate that welfare rights advice services may have been subject to evaluation that had not been widely published. However, our comprehensive search of two academic databases and relevant websites, with additional searching in article reference lists, yielded a high number of studies that were initially subjected to title and abstract screening. We hope that the recommendations we make in this article to support monitoring and evaluation activities will contribute to increased availability and accessibility of evidence to help us to understand service effectiveness.

## 5 CONCLUSION

The review identifies mental health and well‐being impacts attributed to advice service interventions and updates the evidence base to include a decade of significant economic, public health and policy change. However, the evidence to inform the effective delivery of advice services to maximise health outcomes remains scarce. This raises the important question of why the evidence base remains underdeveloped in a decade where the need for robust evidence of how services can address health is strong. Our evidence paints a picture of complexity, from participants with multiple needs, to differing and ambiguous interventions and inconsistent and complicated outcomes. In order to understand this complexity, academic research should embrace systems theory approaches and adopt realist evaluation and logic model methods in a local context. Complexity means it is vital to gather evidence with an overriding awareness of what it means in a local area, within a set of interacting services and within the lives of those who seek advice.

This complexity can be addressed by better connections between academic researchers and advice organisations and commissioners in local areas in order to co‐produce research and to work towards an agreed acceptance of certain forms of evidence. Secondly, the systematic and joined up measuring of outcomes between local organisations and commissioners in order to identify what evidence commissioners are looking for. Thirdly, the co‐location of services, including advice services within primary care locations but also further investment in hub models of delivery that bring together a diverse range of services to address multiple disadvantage and short‐ and long‐term health and wellbeing.

## CONFLICT OF INTEREST

Neither author declares any conflict of interest.

## Supporting information

Supplementary Material

## Data Availability

The data that support the findings of this study are available in the supplementary material of this article.
