# Supplementary material for: Maximising the health impacts of free advice services in the UK: A mixed methods systematic review
Source: Health Soc Care Community. 2022 Mar 21;30(5):1713–25. doi: 10.1111/hsc.13777 (PMC9545623; doi:10.1111/hsc.13777)
Supplement: Supplementary file 1 — Supplementary Material [file HSC-30-1713-s001.docx]

**Supplementary materials**

Additional information is provided on the search strategy used to identify evidence to include in the review, studies excluded from the review, the assessment of methodological quality using the Joanna Briggs Institute’s tools, and the outcomes from individual studies. The completed PRISMA statement is also included.

**Contents**

[1.Search strategy 2](#_Toc96679447)

[2.Excluded studies 3](#_Toc96679448)

[3.JBI Critical Appraisal: Quasi-experimental studies 7](#_Toc96679449)

[4.JBI Critical Appraisal: Qualitative studies 8](#_Toc96679450)

[5.Detail on study outcomes 9](#_Toc96679451)

[6.PRISMA Checklist 13](#_Toc96679452)

## 1.Search strategy

The search strategy used to identify articles in Medline, adapted for use in Social Policy and Practice, is presented along with the list of organisation websites searched for additional studies:

Medline search

| 1 | (TI=(“citizen* advice” OR “advice service*”) ) OR (AB=(“citizen* advice” OR “advice service*”) ) | 201 |
| --- | --- | --- |
| 2 | (TI=((welfare OR “welfare rights” OR legal OR benefits) NEAR/1 (advice OR service OR assistance OR aid))) OR (AB=((welfare OR legal OR benefits) NEAR/1 (advice OR service OR assistance OR aid))) | 4,974 |
| 3 | #1 OR #2 | 5,149 |
| 4 | (TI=(employment OR income OR wage OR debt OR money OR housing OR home* OR accommodation OR mortgage OR health OR wellbeing OR well-being)) OR (AB=(employment OR income OR wage OR debt OR money OR housing OR home* OR accommodation OR mortgage OR health OR wellbeing OR well-being)) | 3,723,603 |
| 5 | #3 AND #4 | 3,612 |
| 6 | Refine by year 2010-2021 | **1,830** |

Last date searched: 13/01/21

Website search

| **Organisation websites searched** | |
| --- | --- |
| [Citizens Advice](https://www.citizensadvice.org.uk/) | [The King's Fund](https://www.kingsfund.org.uk/) |
| CPAG \| | AdviceUK |
| Rightsnet | The Health Foundation |
| Age UK | National Audit Office (NAO) |
| The Joseph Rowntree Foundation | The Low Commission and Legal Support in the UK |
| Advice Services Alliance | Legal Action Group |
| Fuse \| Fuse \| Newcastle University | Healthy Advice |
| Health Education England | Public Health England |
| National Institute for Health Research \| NIHR | HousingCare |
| Financial Capability Strategy for the UK \| Helping people - FinCap | Department of Health and Social Care - GOV.UK (www.gov.uk) |

Last date searched: 29/01/21

## 2.**Excluded** studies

The details of the 58 studies excluded during the screening of full text articles is provided here, organised by the reason they were excluded based upon review inclusion criteria:

**Did not include an evaluation element (30)**

Age UK, 2010. *Promoting mental health and well-being in later life*.

Age UK, 2013. Information and Advice for Older People Evidence Review.

Allmark, P., Baxter, S., Goyder, E., Guillaume, L., and Crofton-Martin, G., 2013. Assessing the health benefits of advice services: Using research evidence and logic model methods to explore complex pathways. *Health and Social Care in the Community*, 21(1), pp.59–68.

Beardon, S. and Genn, H., 2018. *The Health Justice Landscape in England and Wales: Social welfare legal services in health settings*.

Budd, C., 2018. *Advice in practice: Understanding the effects of integrating advice in primary care settings*. London: Citizens Advice.

Caper, K. and Plunkett, J., 2015. *A very general practice*. London: Citizens Advice. Available from: https://www.citizensadvice.org.uk/Global/CitizensAdvice/Public services publications/CitizensAdvice_AVeryGeneralPractice_May2015.pdf.

Carr, S., 2015. *Fusebrief: How do Citizens Advice Bureau services improve people’s health*.

Citizens Advice, 2012. *An overview of possible links between advice and health*.

Citizens Advice, 2015. *Prescribing advice – A pathway to health and saving*.

Citizens Advice, 2020. *Impact Report 2019/20*. Citizens Advice.

Citizens Advice Bureau Scotland, 2015. *Measuring Outcomes from Citizens Advice*. Available from: https://www.cas.org.uk/system/files/publications/measuring_outcomes_from_citizens_advice.pdf.

Corbett, A., Stevens, J., Aarsland, D., Day, S., Moniz-Cook, E., Woods, R., Brooker, D., and Ballard, C., 2012. Systematic review of services providing information and/or advice to people with dementia and/or their caregivers. *International Journal of Geriatric Psychiatry*, 27(6), pp.628–636.

Farr, M. and Cressey, P., 2019. The social impact of advice during disability welfare reform: from social return on investment to evidencing public value through realism and complexity. *Public Management Review*, 21(2), pp.238–263.

ILC, 2016. *What works? A review of the evidence on financial capability interventions and older people in retirement*. International Longevity Centre - UK (ILC-UK). Available from: https://ilcuk.org.uk/wp-content/uploads/2018/10/What-works.pdf.

IPR, 2014. *Proving the value of advice: a study of the impact of Citizens Advice Bureau services*. Bath: Institute for Policy Research.

Kenrick, J., 2011. The outcomes & impact of youth advice – the evidence social welfare advice services. , (December).

Kings Fund, 2019. *What does improving population healthreally mean?*

Langford, K., Baeck, P., and Hampson, M., 2013. More than medicine: New services for people powered health [Online]. *Nesta: Innovation Unit*, p.29. Available from: https://media.nesta.org.uk/documents/more_than_medicine.pdf.

Low Commission, 2015a. *Getting it Right in Social Welfare Law*.

Low Commission, 2015b. The role of advice services in health outcomes: evidence review and mapping study [Online]. , (June), p.97. Available from: https://www.thelegaleducationfoundation.org/wp-content/uploads/2015/06/Role-of-Advice-Services-in-Health-Outcomes.pdf.

Mackenzie, D.G., 2015. Low Commission’s recommendations on welfare advice in healthcare settings are welcome [Online]. *BMJ (Clinical research ed.)*, 351(July), p.h3958. Available from: http://dx.doi.org/doi:10.1136/bmj.h3958.

McDaid, D., La Park, A., and KNapp, M., 2017. *Barriers and Facilitators to Commissioning Cost-Effective Services for Promotion of Mental Health and Wellbeing and Prevention of Mental Ill-Health About Public Health England*.

Mustafa, J., Hodgson, P., Lhussier, M., Forster, N., Carr, S.M., and Dalkin, S.M., 2020. ‘Everything takes too long and nobody is listening’: Developing theory to understand the impact of advice on stress and the ability to cope [Online]. *PLoS ONE*, 15(4), pp.1–22. Available from: http://dx.doi.org/10.1371/journal.pone.0231014.

Paparella, G., 2015. *Debt and health: a briefing*. Available from: http://www.picker.org/wp-content/uploads/2015/12/Debt-and-Health-A-briefing.pdf.

Parsonage, M., 2013. Welfare advice for people who use mental health services: Developing the business case [Online]. *Centre for Mental Health*. Available from: http://www.centreformentalhealth.org.uk/pdfs/Welfare_advice_MH_services.pdf.

Public Health England, 2017. *Commissioning Cost-Effective Services for Promotion of Mental Health and Wellbeing and Prevention of Mental Ill-Health*.

Scottish Government, 2018. *Review of Publicly-funded Advice Services in Scotland*.

Tinelli, M., McDaid, D., Knapp, M., and Guy, D., 2019. *Providing debt advice: economic evidence*.

University of Chester, 2012. *Report into the Added Value of Wirral CAB*. The Centre for Labour Market Development.

Welsh Government, 2013. *Advice Services Review: Final research report*.

**Did not include health or wellbeing outcomes (8):**

Balmer, N.J., Pleasence, P., and Buck, A., 2010. Psychiatric morbidity and people’s experience of and response to social problems involving rights. *Health and Social Care in the Community*, 18(6), pp.588–597.

Forster, N., Dalkin, S.M., Lhussier, M., Hodgson, P., and Carr, S.M., 2016. Exposing the impact of Citizens Advice Bureau services on health: A realist evaluation protocol. *BMJ Open*, 6(1), pp.1–7.

Haighton, C., Moffatt, S., Howel, D., McColl, E., Milne, E., Deverill, M., Rubin, G., Aspray, T., and White, M., 2012. The Do-Well study: Protocol for a randomised controlled trial, economic and qualitative process evaluations of domiciliary welfare rights advice for socio-economically disadvantaged older people recruited via primary health care [Online]. *BMC Public Health*, 12(1), p.1. Available from: ???

Kite, A., 2014. *PhD Thesis: “Citizens Advice in GP Surgeries : an investigation”*. University of Bristol.

Legal Action Group, 2014. *Healthy Legal Advice: Findings from an opinion poll of GPs*. Available from: https://www.lag.org.uk/about-us/policy/research-200550.

Liverpool CCG, 2019. *An exploratory analysis of linked data from health, social care and citizen’s advice case management systems*. Liverpool Clinical Commissioning Group.

SCHE, 2017. *Evaluation of the Tenovus Money Advice Service*.

Woodhead, C., Collins, H., Lomas, R., and Raine, R., 2017. Co-located welfare advice in general practice: A realist qualitative study. *Health and Social Care in the Community*, 25(6), pp.1794–1804.

**Did not evaluate a relevant free to access advice service or intervention (18):**

Balmer, N.J. and Pleasence, P., 2012. *The Legal Problems and Mental Health Needs of Youth Advice Service Users : The Case for Advice*.

Balmer, N.J., Smith, M., Denvir, C., and Patel, A., 2012. Just a phone call away: Is telephone advice enough? *Journal of Social Welfare and Family Law*, 34(1), pp.63–85.

Bradley, L., 2011. Legal support as Enhanced Treatment. *The Strategic Society Centre*, (January).

Braid, B., 2018. Social prescribing: a part of something bigger | The King’s Fund [Online]. *The King´s Funde*, (December 2018), pp.1–3. Available from: https://www.kingsfund.org.uk/blog/2018/12/social-prescribing-part-something-bigger.

Burgess, G., 2010. Housing an ageing population - the value of information and advice. *Housing, Care and Support*, 13(1), pp.19–27.

Eynon, C.A., Robinson, L.J., and Smith, K.M., 2020. Medical–legal partnerships: 11 years’ experience of providing acute legal advice for critically ill patients and their families. *Journal of the Intensive Care Society*, 21(1), pp.40–47.

Ezer, T., Burke-Shyne, N., and Hepford, K., 2018. Legal Support for Palliative Care Patients [Online]. *Journal of Pain and Symptom Management*, 55(2), pp.S157–S162. Available from: https://doi.org/10.1016/j.jpainsymman.2017.03.025.

Gostin, L.O., Monahan, J.T., Kaldor, J., DeBartolo, M., Friedman, E.A., Gottschalk, K., Kim, S.C., Alwan, A., Binagwaho, A., Burci, G.L., Cabal, L., DeLand, K., Evans, T.G., Goosby, E., Hossain, S., Koh, H., Ooms, G., Roses Periago, M., Uprimny, R., and Yamin, A.E., 2019. The legal determinants of health: harnessing the power of law for global health and sustainable development [Online]. *The Lancet*, 393(10183), pp.1857–1910. Available from: http://dx.doi.org/10.1016/S0140-6736(19)30233-8.

Lawton, E. and Tyler, E.T. obi., 2013. Optimizing the health impacts of civil legal aid interventions: the public health framework of medical-legal partnerships. *Rhode Island medical journal (2013)*, 96(7), pp.23–26.

League, A., Donato, K.M., Sheth, N., Selden, E., Patel, S., Cooper, L.B., and Mendenhall, E., 2020. A Systematic Review of Medical-Legal Partnerships Serving Immigrant Communities in the United States [Online]. *Journal of Immigrant and Minority Health*. Available from: https://doi.org/10.1007/s10903-020-01088-1.

Lewis, V., Adamson, L., and Hawthorne, F., 2018. Health justice partnerships: a promising model for increasing access to justice in health services. *Australian Health Review*, 43(6), pp.636–638.

NHS Health Education England, 2016. *Social Prescribing at a Glance*. Available from: https://www.hee.nhs.uk/sites/default/files/documents/Social Prescribing at a glance.pdf.

Pettignano, R., Bliss, L., and Caley, S., 2014. The health law partnership: A medical-legal partnership strategically designed to provide a coordinated approach to public health legal services, education, advocacy, evaluation, research, and scholarship. *Journal of Legal Medicine*, 35(1), pp.57–79.

Regenstein, M., Trott, J., Williamson, A., and Theiss, J., 2018. Addressing social determinants of health through medical-legal partnerships. *Health Affairs*, 37(3), p.378.

Retkin, R., Antoniadis, D., Pepitone, D.F., and Duval, D., 2013. Legal Services: A Necessary Component of Patient Navigation [Online]. *Seminars in Oncology Nursing*, 29(2), pp.149–155. Available from: http://dx.doi.org/10.1016/j.soncn.2013.02.010.

Salter, A.S., Anderson, G.T., Gettinger, J., and Stigleman, S., 2018. Medical-Legal Partnership in Western North Carolina: Addressing Social Determinants of Health Through Team-Based Care. *North Carolina medical journal*, 79(4), pp.259–260.

Sinclair, J., 2017. Building Connections : Glasgow Centre for Population Health. , (December).

Weintraub, D., Rodgers, M.A., Botcheva, L., Loeb, A., Knight, R., Ortega, K., Heymach, B., Sandel, M., and Huffman, L., 2010. Pilot study of medical-legal partnership to address social and legal needs of patients. *Journal of health care for the poor and underserved*, 21(2 Suppl), pp.157–168.

**Study was published prior to 2010 (2):**

Brown, I., 2004. *The role of the Citizens Advice Bureau in supporting health care*. NT Clinical.

Jarman, B., 1985. *Giving advice about welfare benefits in general practice*.

## 3.JBI Critical Appraisal: Quasi-experimental studies

N: No, Y: Yes, U: Unclear, NA: Not Applicable, Ratings: Strong (>81%), Moderate (>61%), Weak (60% and below)

## 4.JBI Critical Appraisal: Qualitative studies

N: No, Y: Yes, U: Unclear, NA: Not Applicable, Ratings: Strong (>81%), Moderate (>61%), Weak (60% and below)

## 5.Detail on study outcomes

This table provides additional detail on the specific outcomes in included studies. A range of outcomes and measures were included in each of the four health and wellbeing categories, and the financial outcomes.

| **Health and wellbeing outcomes** | | | |
| --- | --- | --- | --- |
| **Mental health condition** | **Wellbeing measures** | **Stress** | **Use of healthcare** |
| Includes subjective and validated measures (GMQ-12, HADS) of mental health conditions, including anxiety and depression | Includes subjective and validated measures (WEMWBS, SWEMWBS, CASP-19) of emotional and social wellbeing and quality of life | Includes measures such as PSS and SF-36, as well as subjective measures | Includes measures of number of appointments and prescriptions issues after advice interventions |
| **A reduction in the proportion of individuals meeting criteria for CMD** (GMQ-12) was greater in the advice group compared to controls and this reduction was statistically significant for women and black participants (Woodhead et al 2017a, 2017b)  **Reduced anxiety and depression:** Statistically significant improvements in HADS (Anxiety and Depression) at Wave 2. At Wave 3 statistically significant improvements in HADS (Anxiety). (Jones 2011)  **A reduced depression and anxiety** for 81% in a client survey  (East Staff CAB, 2015)  **Reduced anxiety:** Participants reported reduced anxiety  (Moffatt et al, 2012)  **Addressing the symptoms of mental illness:** Limiting panic attacks and suicidal thoughts  **(**Burrows et al, 2011) | **Increased wellbeing** with **WEMWBS** score of 26.9 before advice and 46.5 after advice  Dalkin et al (2019)  **No evidence of different wellbeing score** **(SWEMWBS)** changes between the groups but sub-group analysis found those with positive advice outcomes had improved wellbeing scores compared to controls.  (Woodhead et al 2017a, 2017b)  **Significant rises in wellbeing** measured on the **WEMWBS** scale, as a result of advice  Farr et al (2014)  **No significant difference in the CASP-19 score (quality of life)** between intervention and control participants at 24 months.  Howel et al (2019)  **Statistically significant improvements in quality of life: SF-36** (Social Functioning, Role Emotional and Mental Health), at Wave 2 and statistically **significant improvements in SF-36** (Vitality, Social Functioning and Mental Health) at wave 3.  Jones, (2011)  **Increased confidence** (majority reported)  (Woodhead et al 2017a, 2017b)  **Decreased social isolation**  Jones, (2011)  (Cooper, 2015)  **Day to day activities**: Better able to enjoy day to day activities  Jones, (2011)  **Improved independence** and capacity to engage in daily activities  (Moffatt et al, 2012)  **Improvements in physical health** reported by 45% in client survey  (East Staff Cab, 2015)  **Health and wellbeing improvements reported by** 68% in their participants  (Boston Citizens Advice, 2012)  **Improved wellbeing** for 448 clients and 'improved health' for 329 clients out of 739 who received casework advice in a three-month period  **And** reported by 93% in participant interviews (n = 44)  (Cooper, 2015)  **Capacity to manage health:** In their client survey 77% reported an increase in capacity to manage own physical and/or mental health  (Kerr et al, 2019)  **positive impact on quality of life**.  Howel et al (2019) | **Reductions in stress** with **PSS** score of 31.4 before advice and 10.3 after advice. Dalkin et al (2019)  **Reported reduced stress**  (Woodhead et al 2017a, 2017b)  **Reduced stress**  Jones, (2011)  **Addressing stress**  (Moffatt et al, 2010)  **Reduced stress**  (Moffatt et al, 2012)  **Reported reduced stress**  (East Staff Cab, 2015) | Overall reduction in GP appointments after advice referrals  (Krska et al, 2013; NHS Sefton, 2010)  A small increase in number of appointments relating to mental health and referrals to mental health services after advice referrals  (Krska et al, 2013; NHS Sefton, 2010)  A “small but significant” reduction in the prescription of hypnotics and anxiolytics (42%) and a reduction in the prescription of antidepressants (22%) after advice was received.  (Krska et al, 2013; NHS Sefton, 2010)  No change in 3-month GP consultation rates amongst advice participants  (Woodhead et al, 2017a) |

| **Financial outcomes** | | |
| --- | --- | --- |
| **Income gains** | **Debt managed or written off** | **Other financial outcomes** |
| Refers to the amount of income that has been increased for individuals as a result of advice, usually in a year period, or as an average amount per person. | Refers to the amount of debt that has been managed or written off for individuals as a result of advice. | Refers to other expressions of financial benefit to the individual as a result of advice. |
| £4,545,623  (Burrows et al, 2011)  £5,392,010  (Moffatt et al, 2012)  £793,135  (Woodhead et al, 2017a)  £508,066  (Farr et al, 2014)  £92,625.01  (Jones, 2011)  £5,002,652.20  (Boston Citizens Advice, 2012)  £6,700,000  (Kerr et al, 2019)  £1,025,575  (East Staff CAB, 2015) | £7.660.593  (Burrows et al, 2011)  £374,502.84  (Jones, 2011)  £2,700,000  (Kerr et al, 2019)  £9383  (East Staff CAB, 2015) | **Improved financial situation:** 164 clients out of 739 who received casework advice and support in a three-month period (Apr-Sep 2015) reported an ‘improved financial situation’ (Cooper, 2015)  **Financial strain:** less common in advice group compared to controls. **Financial support seeking:** A significant impact of advice on reduced use of a credit card or overdraft. **Improved financial situation** reported by advice group but not by comparison group. (Woodhead et al, 2017a, 2017b) |

| **Delivery and implementation outcomes** | |
| --- | --- |
| **Accessibility** | **Satisfaction** |
| Refers to outcomes that relate to the accessibility of advice services. | Refers to outcomes that relate to the satisfaction with advice services reported by external staff and service users. |
| **Accessibility:** "Key positive service features reported by all groups were: the confidential, non-stigmatizing and familiar environment of a general practitioner’s (GP) surgery; the ability to make appointments and experienced advisor availability and continuity."  **(Burrows et al, 2011)**  **Accessibility:** Nearly all participants expressed a preference for GP-located advice because it was easier to access (for example because of mobility problems or travel anxiety), nearer home or more convenient (129, 64.8%), or because it was a more familiar or less anxiety provoking environment (42, 21.2%). If GP-located advice was not available, almost half would have spoken to their GP about their non-health issue or not sought advise at all. (**Woodhead et al, 2017a, 2017b)** | **Satisfaction:** "High levels of satisfaction from service users and referrers”  **(Kerr et al, 2019)**  **Satisfaction:** “The CABHO service is viewed positively by practice managers and GPs and is perceived to benefit both their patients and the practices.”  **(NHS Sefton (2010)**  **Satisfaction:** "99% satisfaction from people who have used the service, and 99% would recommend it to others."  **(Cooper, 2015)**  **Satisfaction:** in their satisfaction survey 95% of their clients reported “overall satisfaction” with the service they received and 96% were satisfied with the 'adviceline' telephone service  **(East Staff CAB, 2015)** |

## 6.PRISMA Checklist

| **Section and Topic** | **Item #** | **Checklist item** | **Location where item is reported** |
| --- | --- | --- | --- |
| **TITLE** | | |  |
| Title | 1 | Identify the report as a systematic review. | Title (p.1) |
| **ABSTRACT** | | |  |
| Abstract | 2 | See the PRISMA 2020 for Abstracts checklist. | Abstract (p.1) |
| **INTRODUCTION** | | |  |
| Rationale | 3 | Describe the rationale for the review in the context of existing knowledge. | Introduction (pp.2-3) |
| Objectives | 4 | Provide an explicit statement of the objective(s) or question(s) the review addresses. | Introduction (p.3) |
| **METHODS** | | |  |
| Eligibility criteria | 5 | Specify the inclusion and exclusion criteria for the review and how studies were grouped for the syntheses. | Methods: inclusion criteria (p.5) |
| Information sources | 6 | Specify all databases, registers, websites, organisations, reference lists and other sources searched or consulted to identify studies. Specify the date when each source was last searched or consulted. | SM 1: Search strategies |
| Search strategy | 7 | Present the full search strategies for all databases, registers and websites, including any filters and limits used. | SM 1: Search strategies |
| Selection process | 8 | Specify the methods used to decide whether a study met the inclusion criteria of the review, including how many reviewers screened each record and each report retrieved, whether they worked independently, and if applicable, details of automation tools used in the process. | Study selection (p.4) |
| Data collection process | 9 | Specify the methods used to collect data from reports, including how many reviewers collected data from each report, whether they worked independently, any processes for obtaining or confirming data from study investigators, and if applicable, details of automation tools used in the process. | Data extraction and quality assessment (p.4) |
| Data items | 10a | List and define all outcomes for which data were sought. Specify whether all results that were compatible with each outcome domain in each study were sought (e.g. for all measures, time points, analyses), and if not, the methods used to decide which results to collect. | Inclusion criteria (pp.3,4) |
|  | 10b | List and define all other variables for which data were sought (e.g. participant and intervention characteristics, funding sources). Describe any assumptions made about any missing or unclear information. | Inclusion criteria (pp.3,4) |
| Study risk of bias assessment | 11 | Specify the methods used to assess risk of bias in the included studies, including details of the tool(s) used, how many reviewers assessed each study and whether they worked independently, and if applicable, details of automation tools used in the process. | Data extraction and quality assessment (pp.4,5) |
| Effect measures | 12 | Specify for each outcome the effect measure(s) (e.g. risk ratio, mean difference) used in the synthesis or presentation of results. | N/A |
| Synthesis methods | 13a | Describe the processes used to decide which studies were eligible for each synthesis (e.g. tabulating the study intervention characteristics and comparing against the planned groups for each synthesis (item #5)). | Data extraction and quality assessment, synthesis (pp.5,4) |
|  | 13b | Describe any methods required to prepare the data for presentation or synthesis, such as handling of missing summary statistics, or data conversions. | N/A |
|  | 13c | Describe any methods used to tabulate or visually display results of individual studies and syntheses. | Synthesis (p.5) |
|  | 13d | Describe any methods used to synthesize results and provide a rationale for the choice(s). If meta-analysis was performed, describe the model(s), method(s) to identify the presence and extent of statistical heterogeneity, and software package(s) used. | Synthesis (p.5) |
|  | 13e | Describe any methods used to explore possible causes of heterogeneity among study results (e.g. subgroup analysis, meta-regression). | N/A |
|  | 13f | Describe any sensitivity analyses conducted to assess robustness of the synthesized results. | N/A |
| Reporting bias assessment | 14 | Describe any methods used to assess risk of bias due to missing results in a synthesis (arising from reporting biases). | N/A |
| Certainty assessment | 15 | Describe any methods used to assess certainty (or confidence) in the body of evidence for an outcome. | Study quality (p.9) |
| **RESULTS** | | |  |
| Study selection | 16a | Describe the results of the search and selection process, from the number of records identified in the search to the number of studies included in the review, ideally using a flow diagram. | Figure 1: flow chart of study selection (p.6) |
|  | 16b | Cite studies that might appear to meet the inclusion criteria, but which were excluded, and explain why they were excluded. | SM 2. Excluded studies |
| Study characteristics | 17 | Cite each included study and present its characteristics. | Summary of included articles (pp. 8,9) |
| Risk of bias in studies | 18 | Present assessments of risk of bias for each included study. | SM 3,4 JBI critical appraisal |
| Results of individual studies | 19 | For all outcomes, present, for each study: (a) summary statistics for each group (where appropriate) and (b) an effect estimate and its precision (e.g. confidence/credible interval), ideally using structured tables or plots. | The impact of advice services (pp.9-12), SM 5 Detail on study outcomes |
| Results of syntheses | 20a | For each synthesis, briefly summarise the characteristics and risk of bias among contributing studies. | Table 1: Summary of included articles (pp.7,8), study quality (p.9) |
|  | 20b | Present results of all statistical syntheses conducted. If meta-analysis was done, present for each the summary estimate and its precision (e.g. confidence/credible interval) and measures of statistical heterogeneity. If comparing groups, describe the direction of the effect. | N/A |
|  | 20c | Present results of all investigations of possible causes of heterogeneity among study results. | N/A |
|  | 20d | Present results of all sensitivity analyses conducted to assess the robustness of the synthesized results. | N/A |
| Reporting biases | 21 | Present assessments of risk of bias due to missing results (arising from reporting biases) for each synthesis assessed. | N/A |
| Certainty of evidence | 22 | Present assessments of certainty (or confidence) in the body of evidence for each outcome assessed. | The Impact of advice services (pp.9-12) |
| **DISCUSSION** | | |  |
| Discussion | 23a | Provide a general interpretation of the results in the context of other evidence. | Discussion (pp.12-15) |
|  | 23b | Discuss any limitations of the evidence included in the review. | Discussion (pp.12-15) |
|  | 23c | Discuss any limitations of the review processes used. | Discussion (pp.12-15) |
|  | 23d | Discuss implications of the results for practice, policy, and future research. | Discussion (pp.12-15) |
| **OTHER INFORMATION** | | |  |
| Registration and protocol | 24a | Provide registration information for the review, including register name and registration number, or state that the review was not registered. | N/A |
|  | 24b | Indicate where the review protocol can be accessed, or state that a protocol was not prepared. | Introduction (p.3) |
|  | 24c | Describe and explain any amendments to information provided at registration or in the protocol. | Protocol |
| Support | 25 | Describe sources of financial or non-financial support for the review, and the role of the funders or sponsors in the review. | Title page |
| Competing interests | 26 | Declare any competing interests of review authors. | Title page |
| Availability of data, code and other materials | 27 | Report which of the following are publicly available and where they can be found: template data collection forms; data extracted from included studies; data used for all analyses; analytic code; any other materials used in the review. | Data available upon request |
